# Surgical Reconstruction Options for a Case of Near Total Intestinal Aganglionosis

**DOI:** 10.7759/cureus.31181

**Published:** 2022-11-07

**Authors:** Raymond I Okeke, Christian Saliba, Diana Fan, Justin Lok, Catherine O'Leary, Maaria Chaudhry, Justin Sobrino, Shin Miyata, Christopher Blewett

**Affiliations:** 1 General Surgery, SSM Health Saint Louis University Hospital, Saint Louis, USA; 2 Pediatric Surgery, SSM Health Cardinal Glennon Children's Hospital, Saint Louis, USA; 3 Medicine, Saint Louis University School of Medicine, Saint Louis, USA

**Keywords:** transanal pull through procedure, bowel obstruction, congenital aganglionosis, colonic aganglionosis, aganglionosis

## Abstract

Hirschsprung’s disease is a congenital anomaly affecting neural crest cell migration and proliferation in the myenteric plexi resulting in dysmotility, which can present as bilious emesis, delayed meconium passage, and bowel obstruction in neonates, or chronic constipation in older children. Depending on the extent of aganglionosis, this disease can involve the whole gut. Treatment involves a temporary ostomy and interval definitive surgical reconstruction. In patients with near or total intestinal aganglionosis, however, there is no consensus on the most effective surgical reconstruction as consideration of the length and function of the normal remnant bowel create concerns for complications with short bowel syndrome post-operatively. We present a case of near-total intestinal aganglionosis highlighting the various options for definitive surgical reconstruction.

## Introduction

Hirschsprung’s disease (HD) involves the absence of ganglionic cells in the submucosal and myenteric neuronal plexi [1,2]. It is a neurocristopathy with low penetrance and variable expression that determines the length of the aganglionic segment. HD can present in genetic syndromes like Down’s syndrome, congenital hypoventilation syndrome, and Haddad syndrome and gene mutations such as those to the arranged during transfection (RET) gene [2-5]. It occurs in approximately 1 in 5,000 live births with a male predominance (4:1) [3]. Presenting symptoms include delayed passage of meconium, bowel obstruction, and bilious vomiting. HD varies in length of bowel involvement. It involves clinical groups of ultra-short, short, and long-segment diseases [3,4]. Long-segment colonic, total colonic, and small bowel aganglionosis are variations of long-segment disease [3,6,7]. Total colonic aganglionosis (TCA) occurs in approximately 3% to 15% of patients with HD [4]. TCA can present complications such as enterocolitis and intestinal failure associated with liver disease [6,7]. We present a case of near-total intestinal aganglionosis presenting as bilious emesis and intestinal paresis to highlight the considerations for definite treatment of this extensive disease process.

## Case presentation

**Figure 1:**
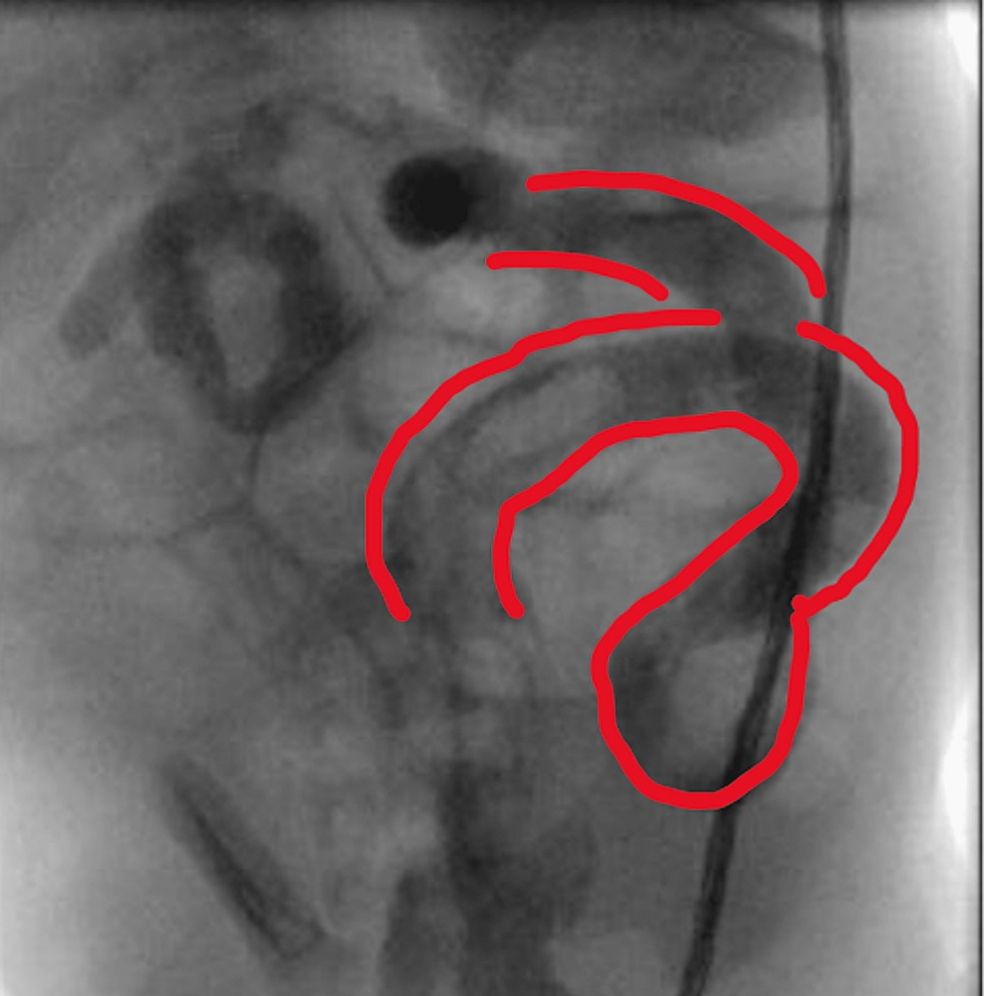
Colon showing “question mark” sign (red outline)

**Figure 2:**
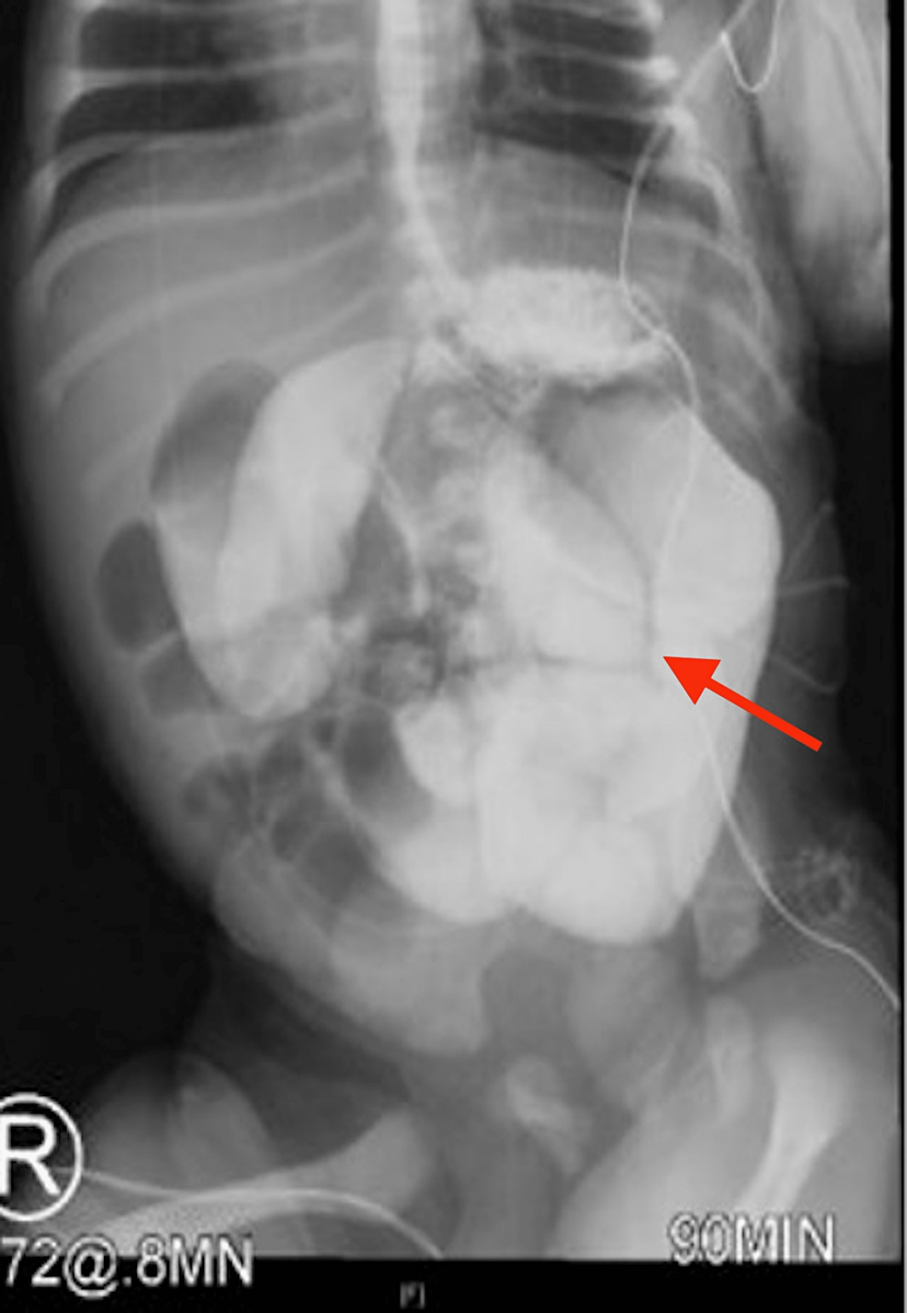
Upper gastrointestinal series showing dilated small bowel loops (red arrow)

The patient is a term 3,300g infant born to a mother with preeclampsia who received good prenatal care. The patient underwent an uneventful vaginal birth and passed meconium on the day of life (DOL) 1 but started with bilious emesis DOL 2. A contrast workup showed a question mark colonic sign (Figure 1) and dilated loops of the small bowel (Figure 2) but revealed no malrotation or volvulus. The patient failed a trial of nasogastric decompression with continued emesis and small bowel distension. We performed an exploratory laparotomy on DOL5. We resected a distal ileal stricture and created an ileostomy and mucous fistula. Histopathology from resected specimen revealed the absence of ganglia to the submucosal and myenteric plexuses at the level of the ileum concerning TCA. Despite conservative management with irrigation three times daily, the patient continued with a high nasogastric tube and low ostomy output. This failure warranted another trip to the OR at three weeks to determine the level of ganglionic bowel. Following multiple circumferential full-thickness bowel biopsies, we found a transition zone at 35 cm from the Treitz ligament with proximal ganglionic cells. We placed a gastrostomy tube and created an end jejunostomy. In standard fashion for TCA involving the small bowel, we performed a subtotal enterectomy [7]. The aganglionic colon was preserved for future surgical reconstruction options.

The patient was started on tube feeds and received cholestyramine, loperamide, and esomeprazole to manage ostomy output. We placed a tunneled catheter for long-term total parenteral nutrition (TPN) at age two months. We discharged the patient at four months on TPN and tube feeds to be followed outpatient by the intestinal rehabilitation and feeding team. The patient was readmitted briefly at ten months for nonbilious non-bloody emesis and lethargy, which improved with rectal irrigations. The patient did not have diarrhea, abdominal pain, or fever. Currently, the patient continues to make some progress in growth and development. The patient can crawl and push to stand, place toys in cups, and wave when prompted. The patient has no oral aversion to food and continues to gain weight appropriately but cannot take steps yet. The patient does not speak words yet but makes a variety of consonants. Our plan will be a re-evaluation at the one/one and a half year of life mark to evaluate for possible reconstruction.

## Discussion

HD presents as functional intestinal obstruction from the incomplete migration of neuroblasts from the neural crest and inadequate differentiation of neuroblasts to ganglion cells in the intestine [4]. TCA involving the small bowel (TCASB) occurs in 2%-5% of HD [8,9]. Like shorter segment HD, TCA may present with symptoms of bowel obstruction, bilious emesis, or failure to pass meconium. TCASB has an overall mortality of 2% to 10% due to complications of obstructive Ileus, short bowel syndrome, and Hirschsprung’s associated enterocolitis (HAEC) [7]. Near total intestinal aganglionosis occurs when the amount of small ganglionic bowel is 40 cm or less [10]. Total intestinal aganglionosis involves the entire gut, including the stomach [10,11]. Our case was diagnosed with near total intestinal aganglionosis with 35 cm of ganglionic bowel distal to the ligament of Treitz.

TCASB requires intra-operative circumferential biopsies at different colon and small intestine levels to determine the extent of aganglionosis [3,7,9]. However, the presence of microcolon and question mark-shaped colon on contrast enema can aid in suggesting the presence of TCA and should be included in the workup when possible [3,9]. In our case, indications for repeat surgery were the absence of ganglion cells in the ileal specimen and continued symptoms of intestinal failure postoperatively. We then made an intraoperative diagnosis of TCASB. We proceeded with small bowel resection after our intraoperative diagnosis as recommended in cases of TCASB [7]. This is performed to reduce the chances of enterocolitis in the excluded aganglionic segment in diverted patients [7]. This form of enterocolitis has been identified in patients with TCASB in addition to enterocolitis at birth before diversion and after the pull-through procedure. In anticipation of a future pull-through procedure, we chose to leave the aganglionic colon in place. Existing literature recommends completing an enterocolectomy of the excluded bowel between 18 months and two years for best outcomes [6]. A newborn can continue conservative treatment if symptoms improve until leveling procedure and surgical reconstruction despite radiographic imaging suggesting TCA and rectal biopsy diagnosing the absence of ganglion cells [12]. The leveling procedure is one in which the level of ganglionic bowel is identified by sequential biopsies from known aganglionosis toward the ligament of Treitz until ganglion cells are identified [7]. A diverting enterostomy is then created at this level. Although this diverting ostomy treats the obstructive symptoms, complications such as dehydration due to persistent diarrhea and long-term fistula have been observed in neonate TCA patients [6,12]. Postoperative complications like enterocolitis and perianal excoriation are problems that may even require re-operations [3,6,9,12]. Liver disease compromises long-term survival without transplantation [6].

Definitive reconstruction should occur between six months and one year depending on the extent of the disease [13]. A colectomy with a straight ileoanal anastomosis and ileostomy can be performed at presentation if the surgeon preserves the dentate line with an intact anal canal [4,14]. Intestinal diversion, however, should be completed within one month after birth, irrespective of the involved segment or an attempt at definitive reconstruction [13]. The surgeon can perform definitive reconstruction if sufficient ganglionic small bowel is available to restore continuity with a chance of proper bowel function [7,14]. The Swenson procedure, first described in the 1940s, is a pull-through procedure for surgical reconstruction. It involves connecting the normally innervated bowel end-to-end with the anal sphincter with complete rectal resection [3,12]. The Soave procedure modifies the pull-through by leaving a rectal muscular cuff by performing a submucosal rectal resection instead. The Duhamel procedure involved a retro-rectal side-to-side anastomosis to a remnant rectum around the region of the dentate line but with no rectal resection. The extended Martin-Duhamel procedure includes the aganglionic rectum in the anastomosis [5,12]. It effectively creates a longitudinal strip to guide propelled fecal stream down toward the anus [3,11]. Currently, there is no significant advantage of one method over the other [3]. With the extensive bowel resection involved in these procedures, the complications of the resultant short bowel syndrome are a vital consideration in our patient and may make these surgical options suboptimal.

Ziegler et al., in 1993, developed the extended myectomy-myotomy surgical technique for near-total and total intestinal aganglionosis to preserve intestinal resorption even in the absence of propulsive motility [11]. This procedure preserves bowel length as propulsive action from the ganglionic bowel forces digestive content toward the anus through the aganglionic segments. The retained aganglionic bowel may prevent failure to thrive in these patients by continuing nutrient absorption. Given patient survival outcomes for a previously fatal diagnosis, this surgical option may hold some promise for patients such as ours with a short gut. However, postoperative outcomes of gut-induced infection and fulminant respiratory failure resulted in death in one-third of the sample population [11]. We considered this option morbid for our patient and proceeded with the option of subtotal enterectomy [7]. Otabor et al. highlighted a successful TPN wean when they performed an extended myectomy-myotomy in a patient with Haddad syndrome and total intestinal aganglionosis [10]. However, despite this promise, patients with ganglionic jejunum less than 50 cm still have poor prognoses. They are mostly permanently parenteral nutrition dependent and can have liver failure requiring transplantation [15]. Sauvat et al. advocate for intestinal transplantation for total intestinal aganglionosis to overcome this parenteral nutrition dependence [16]. However, the outcomes of that study included intestinal fistulas, intestinal perforation, obstruction, a case of intra-abdominal hemorrhage, and deaths from overwhelming sepsis. They did wean all the surviving patients from TPN and had only one patient with fecal continence problems on follow-up.

## Conclusions

Patients with TCA involving the small bowel can have TPN dependence and short gut syndrome with extensive small bowel resection. In addition, if we do not remove the involved small bowel, they can have intestinal failure-associated liver disease and enterocolitis in that small bowel. Therefore, we believe that patient optimization for the definitive pull-through procedure is the best solution to this dilemma. In our case, the patient continues to meet milestones despite short gut syndrome from small bowel resection.
